# Assessing Mothers’ Postpartum Depression From Their Infants’ Cry Vocalizations

**DOI:** 10.3390/bs10020055

**Published:** 2020-02-06

**Authors:** Giulio Gabrieli, Marc H. Bornstein, Nanmathi Manian, Gianluca Esposito

**Affiliations:** 1 Psychology Program, School of Social Sciences, Nanyang Technological University, Singapore 639818, Singapore; giulio001@e.ntu.edu.sg; 2 Eunice Kennedy Shriver National Institute of Child Health and Human Development, Bethesda, MD 20892, USA; marc.h.bornstein@gmail.com; 3 Institute for Fiscal Studies, London WC1E 7AE, UK; 4 Westat, Rockville, MD 20850, USA; nanmathinanian@westat.com; 5 Lee Kong Chian School of Medicine, Nanyang Technological University, Singapore 308232, Singapore; 6 Department of Psychology and Cognitive Science, University of Trento, 38068 Trento, Italy

**Keywords:** infant cry, postpartum depression, acoustic analysis

## Abstract

Postpartum Depression (PPD), a condition that affects up to 15% of mothers in high-income countries, reduces attention to the needs of the child and is among the first causes of infanticide. PPD is usually identified using self-report measures and therefore it is possible that mothers are unwilling to report PPD because of a social desirability bias. Previous studies have highlighted the presence of significant differences in the acoustical properties of the vocalizations of infants of depressed and healthy mothers, suggesting that the mothers’ behavior can induce changes in infants’ vocalizations. In this study, cry episodes of infants (N = 56, 157.4 days ± 8.5, 62% firstborn) of depressed (N = 29) and non-depressed (N = 27) mothers (mean age = 31.1 years ± 3.9) are analyzed to investigate the possibility that a cloud-based machine learning model can identify PPD in mothers from the acoustical properties of their infants’ vocalizations. Acoustic features (fundamental frequency, first four formants, and intensity) are first extracted from recordings of crying infants, then cloud-based artificial intelligence models are employed to identify maternal depression versus non-depression from estimated features. The trained model shows that commonly adopted acoustical features can be successfully used to identify postpartum depressed mothers with high accuracy (89.5%).

**Data Set License:** CC-BY-NC

## 1. Introduction

Cry is an innate behavior and constitutes the first form of communication newborns use to interact with their caregivers [1]. Similar to speech in adults, cry vocalizations are produced by the vibration of the vocal folds, which are controlled by the Central Nervous System (CNS). Therefore, acoustical analysis of cry can identify pathological conditions associated with the vocal tract, the brain, and the spinal cord, as demonstrated in previous research [2,3]. The functional utility of infant cry is to elicit a response in an infant’s caregiver, but some situations and conditions diminish adults’ sensitivity and responsiveness to cry [4,5,6,7,8]. Mothers who suffer from Postpartum Depression (PPD), a condition that is reported by 10–15% of mothers in high-income countries [9,10], and up to 50% in low- and middle-income countries, reduces the level of stimulation produced by infant cry and decreases mothers’ level of responsiveness to the needs of their children [11,12,13,14]. Infants of depressed mothers are therefore exposed to an increased developmental risk [15], but this is not a uni-directional relation: previous studies have identified a bi-directional relationship between mother and child synchrony and well-being. Brand et al. [16], for example, identified a relation between mothers’ sleep quality and well-being with infants’ crying behavior, cortisol secretion, and sleep patterns.

### 1.1. Postpartum Depression Identification

Postpartum Depression (PPD), a very common childbearing complication that develops after a woman has given birth, is defined as per the DSM-V as a major depression disorder that emerges within 4 weeks following delivery that causes (a) significant distress or impairment in occupational, social, or other important area of functioning, (b) is not attributable to effects of any substance, and (c) is characterized by at least five out of the nine symptoms presented in the diagnostic manual (e.g., depressed mood, insomnia, fatigue, recurrent thoughts of death) [17].

Development of postpartum depression is connected to previous episodes of depression and is more common when paired with other stressful events or in women with a family history of mood disorder [9,18]. Rapid hormonal changes after delivery seem to play a primary role in the development of this disorder [19]. It is worth noting that PPD is a mental state which is not related to cultural factors, family income, and cultural background.

While the percentage of new mothers suffering from postpartum depressive symptoms has decreased during the year [20], it is estimated that 60% of mothers with depressive symptoms receive no treatment or a clinical diagnosis [21]. Accordingly to Ko et al. [21], common treatment barriers are to be found in mothers’ opposition to the treatment and concerns about social stigma as well as problems related to the cost of the treatments, possible transportation or time limitations, and lack of knowledge about where to find treatment and about the importance of this mental illness.

The presence of postpartum depression symptoms in mothers is assessed through questionnaires and structured interviews or investigating different biomarkers that have been demonstrated to reflect the risk of developing PPD. The changes in concentration of hair steroid levels—such as cortisol, progesterone, and cortisone—in hair samples measured during pregnancy and after parturition, for example, can be used to predict the development of PPD symptoms [22,23]. The most adopted self-report questionnaires are the Edinburgh Postnatal Depression Scale, a 10-item questionnaire that uses four-point Likert scale responses [24,25] and the Beck Depression Inventory (BDI-II), a 21-item questionnaire of the presence and related degree of depressive symptoms, consistent with the DSM. An alternative approach is the Structured Clinical Interview per DMS Axis I disorders (SCID), a semi-structured diagnostic instrument which is widely employed in clinical trials, and includes nine modules [26] the evaluation of mood, psychotic, anxiety, eating, obsessive-compulsive, substance use, and sleep disorders [27]. The SCID needs to be administered by clinicians with specific training [28].

In this paper, we propose the usage of machine learning models based on the analysis of infants’ cries to support clinicians in identifying postpartum depression symptoms in mothers. The rationale behind this tool is that by analyzing cry samples an initial estimate of the diagnosis can be performed at little to no cost, and in a limited amount of time. Moreover, because cry recordings can be obtained even by parents themselves, the tool can be used in rural areas. Finally, because the tool is not based on mothers’ responses, it may provide feedback which is not influenced by depressed mothers’ fear of being stigmatized. Such a tool may be used to improve clinical diagnosis and thereby enhance the quality of life of both infants and mothers.

### 1.2. Infant Cry

Infants’ actively regulate acoustic information in their vocalizations to express specific needs. For example, acoustical analysis of cries has been used to identify the stimulus to cry, whether hunger, pain, or discomfort [29]. Similarly, babies vocalize differently according to their health status. Analysis of infants’ cries has shown that specific patterns of cry vocalizations reflect infants’ health status [30]. For example, Sheinkopf et al. [31] found different patterns of acoustical properties of cry vocalizations in infants at risk for ASD compared to vocalizations from a healthy control group, with at risk infants producing both pain and non-pain vocalizations at higher fundamental frequency (F_0_), as compared to the control group. Likewise, Garcia and Garcia [32] successfully employed a feed-forward neural network (97% accuracy) to distinguish between cry samples collected from deaf and normal-hearing infants.

In a typical study, cry vocalizations are elicited in babies using a trigger (e.g., heel prick) and recorded on digital or analog sources [33]. Cry signals are then filtered to remove higher frequency components. Finally, acoustic features are estimated from the signals. Commonly used acoustic features are the Fundamental Frequency (F0), which is the lowest pitch of periodic signals, and its formants (F_1_–F_4_), which are frequency peaks with wavelengths multiples of the fundamental frequency.

Different techniques are used to estimate acoustic features from cry samples, automatically (by means of a peak detection algorithm) or manually (by visual inspection of spectrograms). Estimated features are then compared using statistical methods (to investigate the existence of specific patterns associated with a pathology) or imported to a classifier (to investigate whether those differences are adequately robust to be used to identify a clinical situation reliably). Because of depressed mothers’ reduced sensitivity and reactions to infants’ cries, infants may regulate the frequencies of their vocalization to maximize responses of their caregivers. A limited number of studies have focused on cries of infants of PPD mothers, with the majority focusing not on the acoustical properties, but vocalization patterns in terms of quantity and length. In a study of infants of three and six months of age, Milgrom and colleagues [34] found that three-month-olds of depressed mothers cry for longer period of times during an average day, if compared to infants of healthy mothers. These results suggest that infants may increase the frequency of their cry vocalizations to respond to lack of maternal attention [12,34]. Similar results were found by Miller et al. [35] in a study of the length of distress vocalizations in 6-week-old infants, with the vocalizations of infants of depressed mothers significantly longer than those of same age infants of healthy mothers [36].

Concerning the acoustical properties of cry, a previous study identified significant differences between the vocalizations of infants of depressed and non-depressed mothers, with the first producing vocalization at a significantly higher F_0_ and within a smaller frequency range [12].

On these bases, an analysis of the acoustical properties of cry vocalizations could be used to identify, in a non-invasive way, infants of mothers who suffer from PPD.

### 1.3. Cloud Based Model

Because of depressed mothers’ reduced sensitivity and reactions to infants’ cries, children may regulate the frequencies of their vocalization to maximize the responses of their caregivers. A previous study has identified significant differences between the vocalizations of infants of depressed and non-depressed mothers [12]. Therefore, an analysis of the acoustical properties of their cry vocalization can be used to identify the children of depressed mothers.

Big Data computing is a data science paradigm that is gaining popularity in recent years. It refers to the analysis of multi-dimensional information mining for different purposes, including but not limited to the development of new scientific discoveries, implementation of large scale infrastructures, and advanced business analytics [37]. To deal with the need for fast and scalable computing resources, different companies have designed tools for the mining, storage, and analysis of big data. Amongst those, Google${}^{®}$ created a set of Software as a Service (SaaS) that runs in the cloud that can be used by customers to store and analyze datasets [38,39].

Adoption of cloud-based models in scientific research provides different advantages, including the reduction of computational burdening related to storage and computation, provides for high scalability for additional data, and security through the adoption of Secure Socket Layers (SSL) for the connections and the possibility of encrypting stored data [39,40]. However, advanced models based on cloud resourcing require large amounts of data and therefore may not be suitable for the analysis of physiological measures, especially when the number of samples per class is not balanced. A solution proposed for solving missing data values, called data augmentation, consists of reconstructing missing values in balanced two-way tables. It can be adopted in machine learning to increase the number of analyzable data [41]. A prominent approach is Additive White Gaussian Noise (AWGN), which consists in the creation of new values for a dataset by adding white noise to a copy of the values of the original dataset [42,43]. These methods require fewer computational resources and can be employed to increase the dimension of numeric datasets. The technique is based on the assumption that, given a signal, adding noise that follows a Gaussian distribution to a copy of the original signal, and using both the original and the modified versions as a training element for a classifier, enhances the quality of the classifier itself, making it more noise-resistant [44]. Especially useful to increase the number of samples for deep learning images classification, the technique has been proven to work well to increase the accuracy of different classifiers. Rochac et al. [44], for example, employed additive white Gaussian noise to verify whether the accuracy of an image classifier based on convolutional neural network would benefit from the addition of more samples containing added noise. Not surprisingly, their classifier was almost 20% more accurate when the number of initial samples was increased by 100 times. Similarly, Bjerrum et al. [45] verified the performances of a convolutional neural network for the analysis of near-infrared (NIR) spectral signals with and without the addition of additive white Gaussian noise. Their results showed that by increasing the dimensionality of the dataset using AWGN the model could achieve high accuracy.

### 1.4. Aim and Hypothesis

As proven by a previous study [12], the acoustical properties of cry vocalizations of infants of depressed and non-depressed mothers differ significantly. For this reason, in this study, we investigated the possibility of using cry samples to identify infants of depressed mothers. More specifically, we hypothesized that a cloud computing based model could identify infants of mothers suffering from PPD by using acoustic features estimated from recordings of their cry vocalizations.

## 2. Methods

### 2.1. Analytic Plan

**Figure 1:**
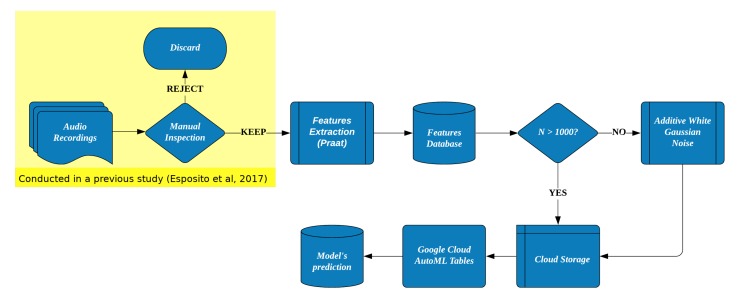
Summary of the steps employed in the development of the model for the diagnosis of Postpartum Depression (PPD) from infants’ cry vocalizations.

In this work, acoustical features (F_0_, F_1–4_, Intensity) were estimated from cry vocalizations collected in a previous study [12]. The full feature extraction procedure is reported in Section 2.3. Then, a cloud-based AI model, based on Google${}^{®}$ AutoML Tables, was trained and tested. A visual representation of the overall process is displayed in Figure 1.

### 2.2. Data

To test our hypothesis, we adopted the recordings from the dataset used in a previous publication on acoustical differences in cry vocalizations of infants of depressed and healthy mothers [12].

Vocalizations from infants of depressed (N = 29, 8 infant girls) and non-depressed mothers (N = 27, 7 infant girls) were collected at home when the infants were about 5 months of age (mean age = 157.4 days ± 8.5). Fifty-six (N = 56) mothers (mean age = 31.1 years ± 3.9) were recruited from the Washington DC metropolitan area by mailing lists and newspaper advertisements; they included European Americans (n = 36), African-Americans (n = 10), Asian Americans (n = 7), American Indians (n = 1), and Latin Americans (n = 2). Concerning their education level, 30% of the mothers completed at least one university graduate program, 50% completed college, while 20% had only partial college education or less. About 60% of infants were first-born, with a percentage slightly higher for infants of depressed mothers (70%). Biological fathers lived with the family at the time of the recordings in all (100%) the households. The study was approved by the IRB of the Eunice Kennedy Shriver National Institute of Child Health and Human Development (protocol code: 02-CH-0278) and was conducted according to the principles expressed in the Declaration of Helsinki. Written informed consent was obtained from all mothers before each recording session.

To increase the ecological validity of data, data were collected in mothers’ homes by researchers of the National Institute of Health (NIH, USA). Mothers were asked to behave as they normally would, ignoring the presence of the experimenters. Infants and mothers were audio and video-recorded for at least 50 min, an amount of time that according to Holden and Millers [46] falls in the optimal time-frame for mother-infant observation.

Mothers’ PPD was assessed using the Structured Clinical Interview for DSM-IV Axis I Disorders (SCID-I) and the Beck Depression Inventory (BDI-II) [47]. Evaluation of the scales was performed by researchers of the National Institute of Health (NIH, USA). Mothers categorized as depressed had a high score on the BDI scale (>12) and had been diagnosed as having minor or major depression (SCID) by the time their infants were five months old.

### 2.3. Features Extraction

Collected cry samples (N = 715) were digitalized in *WAVE* (*wav* file format, two channels) at 44.1 kHz (16 bit). Being a lossless compression format, WAVE has been selected to preserve frequency information convoyed by cry signals, that may have been altered with lossy file formats [33]. Moreover, the sampling rate allows for analysis of frequencies up to 22 kHz, which makes it suitable for reliable analysis up to the fourth formant (F_4_), which is the Nyquist frequency of recorded signal. No further preprocessing was conducted on recorded signals to avoid alterations of frequency information contained within the signal.

Features (F_0–4_) were extracted using Praat (v. 6.0.50, Windows 64 bit), an open-source software designed for voice analysis [48]. This software is based on the spectrographic analysis of a signal by means of a Long-Term Average Spectrum (LTAS), which ensures reliable evaluation of acoustical properties of a signal even in the presence of noise. Specifically, the signal is first segmented into windows of a pre-specified length, then each segment is analyzed utilizing an auto-correlation algorithm that works in the lag-domain (or τ−domain).

Being designed for the acoustical analysis of adult voices, the software’s default settings are not suitable for the analysis of infant cry. To rectify this issue, software settings were adapted to correctly identify F_0_ (lower cutoff = 250 Hz, upper cutoff = 800 Hz) and the first four harmonics (number of harmonics = 5, upper cutoff = 6000 Hz) in a range that covers the spectrum in which infant cry vocalization properties are usually found [49]. A copy of the script used for feature estimation is available online [50].

### 2.4. Classification

To investigate the possibility of using advanced Cloud Computing techniques to verify whether machine learning models could be used to identify infants of depressed mothers, we relied on the Google Cloud Platform${}^{®}$: *Google AutoML Tables* (https://cloud.google.com/automl-tables/) [51]. A binary classification model was employed to discriminate between the cries of infants of mothers suffering from PPD from those of infants of healthy mothers. AutoML Tables were configured so that 80% of imported data was used for training, 10% for validation, and 10% for testing.

The model was executed up to two node hours (total running time of the training phase spread across the different machines that compose a node), while the model was uploaded using server-side encryption. Accuracy of the model was evaluated in terms of Precision (expressed in percentage), Area under the precision-recall curve (AUC PR, a value between 0 and 1, such that the higher the value, the higher the quality of the model), area under the curve of the receiver operative characteristics (AUC ROC, a value between 0 and 1, such that the higher the value, the higher the quality of the model), and logarithmic loss (a value between 0 and 1, such that the lower the value, the higher the quality of the model.

#### Data Augmentation

AutoML Tables require at least 1000 samples to be executed (Beta version), therefore a data augmentation technique, Additive White Gaussian Noise (AVGN), was applied to increase the number of samples of the dataset.

In this study, AWGN (±1 STD) was applied to the acoustical features’ values of a copy of the dataset and then merged with the original samples to obtain a dataset about twice the size of the original set of data (N = 1413). Augmented dataset, containing both acoustic (F_0_, F_1–4_, and Intensity) and demographic information (infants’ gender, mothers’ age) was employed for classification purposes. A copy of the final dataset is available online in the data repository of the Nanyang Technological University [50].

## 3. Results

Model training stopped after 0.916 node hours, reporting an average accuracy on the test set of 89.5%, as well as robust values for AUC PR (0.954), AUC ROC (0.969), and logarithmic loss (0.250). Overall, the model achieved more than the 90% of precision (90.4%), with a true positive recall of 88.8% and an almost null false positive rate (0.09). Metrics of the score of the different evaluations are reported in Table 1.

For the model’s error distribution, the confusion matrix of the model is reported in Table 2.

## 4. Discussion

In this work, we tested the possibility of using machine learning models to identify postpartum depression in mothers from characteristics of their infants’ cry vocalizations.

Results of our model, trained on Google${}^{®}$’s cloud computing service, demonstrate the robustness of the method based on analysis of infants’ cries. More specifically, by using commonly investigated acoustical properties of cry vocalizations, our model identified, with a high degree of accuracy (89.5%) the vocalizations produced by infants of depressed mothers. This is especially important if we consider that a model trained on AutoML Table can be easily integrated with web or mobile applications and can, therefore, be useful to those who have limited or no access to health services and clinical supports. Moreover, releasing the model as a software may allow us to obtain more samples that, when combined with proper clinical evaluation, will increase the accuracy of the model and reduce the risk of overfitting.

Additionally, our results support the work from Esposito et al. [30], confirming the presence of acoustical differences in the vocalizations of infants of depressed and non-depressed mothers, as well as suggesting the reliability of the Data Augmentation technique for the analysis of cry vocalizations using machine learning models.

Our results suggest that machine learning models, trained in cloud environments, can support clinicians in the diagnosis of PPD.

### Limitations

Despite these promising results, some limitations need to be addressed. First, our model was tested on a single dataset, which was expanded using a data augmentation procedure. Future studies should address the performance of models on data collected from different participants to verify the broader utility of the methods. Moreover, we trained the models using only acoustical features and demographic information about mothers (age) and infants (gender). Future studies might also address how including additional data, such as questionnaire scores (BDI) or the gestational ages of the babiesat birth, might improve predictive models by reducing the ratio of false positives and false negatives.

Additionally, special attention has to be drawn to privacy issues in investigating health-related problems on the cloud. In our study, the data of multiple participants were anonymously imported to the model, making it impossible to match model predictions with participant demographic information. In a real application of the technique, special measures would have to be taken to prevent any possible leak of data that could undermine patient privacy and well-being.

Finally, because of the fact that our analysis is based on data collected on a previous study, and because of the limitations of the technology then used, we are not able, at this stage, to provide a reliable investigation of how the level of depression affects the accuracy of the model, as this would require not only a greater number of samples but also a balanced distribution of levels of depression of participating mothers.

## 5. Conclusions

In this study, we investigated the possibility of using cloud-based machine learning models to identify postpartum depression in new mothers by analyzing their infants’ cry vocalizations. By employing a machine learning model based on Google’s Cloud Platform${}^{®}$, we demonstrated that, by using acoustical features estimated from cry recordings, it is possible to identify, with a good degree of accuracy (89.5%), the vocalization produced by infants of depressed mothers.

Despite our relatively small sample size, and the fact that the dataset was not originally designed for this kind of analysis, our results are promising for the development of a low-cost tool that can be employed by clinicians to support their diagnosis of PPD.

Future studies should address whether similar models can obtain better performances by studying larger numbers of vocalizations collected from different infants from a more variegated population to overcome possible problems because the participants in our study were all located within a small geographical area [52]. Additionally, future work should verify whether a different set of features, such as those based on the cepstrum analysis, could be used to enhance the performances of machine learning classifiers.

## Figures and Tables

**Table 1:** Google’s AutoML Model Evaluation Metrics.

Metric	Score
AUC PR	0.954
AUC ROC	0.969
Logarithmic Loss	0.250
Accuracy	89.5%
Precision	90.4%
True positive rate (Recall)	88.8%
False positive rate	0.090

**Table 2:** Google’s AutoML Model Confusion Matrix.

	Predicted Label	
True Label	False	True
False	88%	12%
True	9%	91%

## Abbreviations

The following abbreviations are used in this manuscript:

PPD	Postpartum Depression
CNS	Central Nervous System
DSM	Diagnostic and Statistical Manual
SCID	Structured Clinical Interview
SaaS	Software as a Service
SSL	Secure Sockets Layer
AWGN	Addittive White Gaussian Noise
LTAS	Long-Term Average Spectrum
AUC PR	Area Under the Curve: Precision-Recall
AUC ROC	Area Under the Curve: Receiver Operative Characteristics
